# Modeling Patient Treatment With Medical Records: An Abstraction Hierarchy to Understand User Competencies and Needs

**DOI:** 10.2196/humanfactors.6857

**Published:** 2017-07-28

**Authors:** Justin D St-Maurice, Catherine M Burns

**Affiliations:** ^1^ Systems Design University of Waterloo Waterloo, ON Canada; ^2^ Applied Health Information Science School of Health & Life Sciences and Community Services Conestoga College Institute of Technology and Advanced Learning Kitchener, ON Canada

**Keywords:** clinical decision-making, health services research, qualitative research, primary health care, medical records systems, computerized

## Abstract

**Background:**

Health care is a complex sociotechnical system. Patient treatment is evolving and needs to incorporate the use of technology and new patient-centered treatment paradigms. Cognitive work analysis (CWA) is an effective framework for understanding complex systems, and work domain analysis (WDA) is useful for understanding complex ecologies. Although previous applications of CWA have described patient treatment, due to their scope of work patients were previously characterized as biomedical machines, rather than patient actors involved in their own care.

**Objective:**

An abstraction hierarchy that characterizes patients as beings with complex social values and priorities is needed. This can help better understand treatment in a modern approach to care. The purpose of this study was to perform a WDA to represent the treatment of patients with medical records.

**Methods:**

The methods to develop this model included the analysis of written texts and collaboration with subject matter experts. Our WDA represents the ecology through its functional purposes, abstract functions, generalized functions, physical functions, and physical forms.

**Results:**

Compared with other work domain models, this model is able to articulate the nuanced balance between medical treatment, patient education, and limited health care resources. Concepts in the analysis were similar to the modeling choices of other WDAs but combined them in as a comprehensive, systematic, and contextual overview. The model is helpful to understand user competencies and needs. Future models could be developed to model the patient’s domain and enable the exploration of the shared decision-making (SDM) paradigm.

**Conclusion:**

Our work domain model links treatment goals, decision-making constraints, and task workflows. This model can be used by system developers who would like to use ecological interface design (EID) to improve systems. Our hierarchy is the first in a future set that could explore new treatment paradigms. Future hierarchies could model the patient as a controller and could be useful for mobile app development.

## Introduction

Health care is considered a complex sociotechnical system [1]. Additionally, there is a trend to move away from paternalistic health care approaches to treatment [2,3] and engage patients in their own care. For example, there is currently a trend to adopt shared decision making (SDM) [4,5] to improve patient care through engagement. Similarly, new health care laws are promoting patient-centered care as a priority paradigm shift (eg, the Ontario’s Patients First Act). As the health care delivery environment incorporates new constraints and develops new goals, clinicians have unique needs and require a rich set of competencies to practice medicine. As a complex sociotechnical system, using the cognitive work analysis (CWA) framework can be an effective approach to understand and describe the complexities of care in this challenging world.

### Cognitive Work Analysis

CWA is a conceptual framework that facilitates the analysis of complex systems at various levels of detail. It can help assess how the environment impacts and shapes human-information interactions [6]. Work domain analysis (WDA) is the first step of CWA that focuses on ecology. It can provide valuable information about the structure of work, articulate abstract concepts such as professional values and culture, and describe the constraints that operate in the work domain. WDA can describe how structures, abstract values, and constraints affect the normal functions of a system [7]. There are many examples of using CWA in health care [1,8-14].

The abstraction hierarchy (AH) is a modeling tool that describes the results of a WDA [6]. The AH is intended to be a full depiction of the necessary constraints that need to be taken into consideration in order for the system to achieve its purpose, while describing the system’s underlining ecology and limitations [15]. Using AHs can help bridge the psychology-culture-medicine gap in health care. These hierarchies can be used to develop representations of patient care that align with biomedical knowledge, support medical problem solving, and act as a frame of reference [16]. As a structured approach to WDA, the AH includes a layer to describe the system’s functional purposes, abstract functions, generalized functions, physical functions, and physical forms. Lines are shown between each layer to show means-end or how-why relationships [15].

### Work Domains, Patients, and Patient Care

Many AHs have been developed to describe patient health. Some of these AHs were developed through a WDA, whereas others were developed within the context of a fuller CWA exercise. Some of these abstractions treat patients as biomedical machines with physiological processes [16-20]. For example, some models represent the human body in its resting state during anesthesia [19], decompose the human body into systems and organs [10], or describe the cardiovascular system as an independent system [20]. The scope of these analyses is more biomedical in nature because they describe treatments and procedures, and are modeling biomedical treatments after the consultation phase. This scope makes sense within the confines of emergency or surgical care when patients are unconscious; naturally patient values and personal wishes fall out of the scope of such an analysis. In these contexts, “aberrations in physiological and biological regulatory processes” are the “domain upon which clinicians work” [17].

In other cases, patients are conscious and therefore capable participants in their own health care. Ashoori and Burns [12] modeled the patient-as-an-actor approach effectively during a study of a birthing unit. The CWA showed rich coordinative points, shared artefacts and adjusting structures, and described the patient as an active partner that engaged in their own health. In particular, the AH modeled the patient as a physical function of prescription, assessment, and consulting. In another example, Rezai and Burns [13] modeled patient values, skills, support systems, and abilities in a home health care scenario with WDA and Control Task Analysis (ConTA). The scope did not include the patient within clinical practice. Regardless, both examples demonstrate that CWA is capable of characterizing patients as emotionally complex, social creatures, and that CWA can successfully describe patients as decision makers with rich sets of values and capabilities to support their own health care. Within CWA, WDA can describe many complex relationships that are both biomedical and patient-related.

Building a model of patient treatment is challenging, and it is further complicated by the nuances of effectively treating patients with the assistance of electronic medical records (EMRs). At present, no CWA models or AHs of patient treatment address this context and need.

### Model Objective and Scope

The objectives for the AH was to capture the complexities, balances, and challenges regarding patient treatment from a clinician’s perspective. Such a model could be specific to an individual physician, practice, or specialty. In an effort to offer a breadth of utility, the goal of this AH is to capture generic and common health care processes and priorities, without worrying about specific or unusual use cases. The goal is to develop a model that could represent all types of clinicians involved in providing and triaging care.

To represent current and modern approaches to patient treatment, the model must go beyond the laymen’s and paternalistic impression of medicine as a purely biomedical process. Whereas physicians are experts in disease, patients are experts in their own experience of disease and in their preferences [3]. One of the challenges of patient care is incorporating the patient’s values and preferences into decision making [5]. The model must articulate the challenges of treating patients in a modern world that is subject to contradictory sources of health information, conflicting personal values, and complex determinants of health. In this sense, the model needs to describe the biopsychosocial constraints and nuances of patient treatment in the Internet age.

Finally, the model must capture the impact and role of EMRs in delivering health care. The model needs to describe the complex processes associated with using EMR records and how they interact with clinical practice.

### Intended Uses

As an insightful model of patient treatment, the AH should serve several purposes. The goal of developing this AH was to support many use cases, including the following.

#### Change Management

The AH should provide greater context when trying to plan for the implementation of new systems, new processes, and new workflows.

#### EMR Development

As patient treatment complexities evolve, so must EMRs. Providing a better context and understanding of patient treatment could offer valuable insights to EMR developers. Developing a current and modern model of patient treatment addresses a gap and could lead to the design of improved EMR systems.

#### Additional AHs

Clear value is seen in understanding health care as a complex system. An AH of patient treatment could provide a basis for additional analysis. For example, understanding patient treatment would be a precursor to understanding the management of health information and data.

## Methods

### Study Context

The study was conducted through collaborations with subject matter experts (SMEs) such as managers and clinicians in Ontario. The intent of the model was to capture patient treatment in a general way that could encompass different types of patient care situations. To capture a broad set of ideas and clinical processes, SMEs who worked at medium-sized hospitals within primary care clinics and within family health teams were interviewed. The concepts that were included in the model are reflections of a single-payer system in Canada and reflect a Canadian perspective on social determinants of health [21]. The scope of the study included the development of a model to represent all types of clinicians including, but not limited to, physicians, physiotherapists, nurse practitioners, dietitians, mental health workers, and pharmacists.

### Information Gathering and Validation

The development of the AH took place over the span of 12 months. As an initial step, information for our WDA was collected by reviewing textbooks (such as pathophysiology textbooks [22] and health system textbooks [23]), best practice guidelines, professional standards [24,25] and literature [1,4,26-33]. Insightful information and anecdotes were also gathered during previous research [34].

After reviewing literature, we collaborated with 8 SMEs, iterated through various model concepts, re-interviewed SMEs to collect additional feedback and reworked the model as required. Our strategy was to link concepts in the WDA to comments from SMEs. In addition to gathering input from a large group of SMEs, a family physician volunteered to provide feedback after seeing several drafts. While practicing over the course of a week, this physician took notes about the AH and verified that all important concepts, processes, and decision-making tasks he experienced throughout a week were generally included in the model. This helped confirm the insight we collected from the larger group of SMEs. There were 10 iterations and versions of the AH before the development of the final version.

### Abstraction Hierarchy Development

The development of AHs is challenging because there are many ways to model abstract concepts and ideas. Practitioners need to engage and observe users and articulate thoughts and suggestions into the AH. Often, the literal suggestions and ideas from SMEs need to be abstracted into high level concepts and ideas. AHs are intended to be helpful, but not perfect, and managing the scope and level of detail of the modeling exercise is a challenge in itself.

The first phase of a WDA is to determine the system boundary. There is a balance to achieve in the analysis: a domain boundary that is too narrow will leave out connections and interactions that exist outside the boundary, whereas a broad boundary can distract the modeling effort as time is spent developing concepts that are not germane to the modeling objective [10]. As the operator of the system domain was the patient’s clinician, the boundary was restricted to activities that were within the clinician’s control during a patient’s use of services, even if they were indirect. The patient and their attributes were included in the scope of the analysis. Patient flows and activities outside of an encounter with a clinician were excluded from the scope (eg, patient opting not to take medicines, choosing to perform exercises, adjusting diet, and consulting with family).

**Figure 1:**
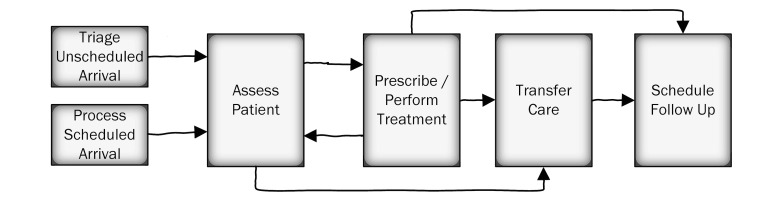
General patient workflow functions.

**Figure 2:**
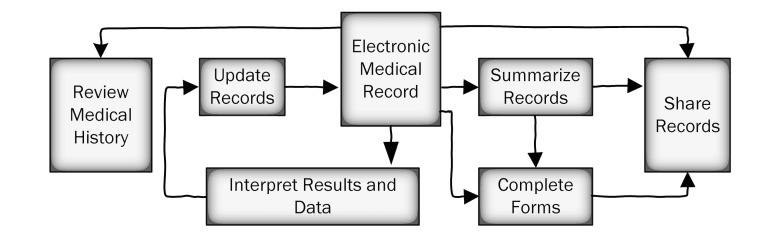
General information workflow functions.

Workflows representing patient flows (see Figure 1) and information processes (see Figure 2) were developed with SMEs to describe the generalized activities of the clinic and its clinicians. As generalized workflows, not all components of the workflow are necessarily “activated” during each patient encounter but represent possible workflows during a visit. In the case of patient flows, most use cases within the clinic (involving a combination of triage, assessment, treatment, care transfer, and scheduling) were captured. In the case of information flows, most use cases involving the EMR (involving a combination of summarization, sharing, updating and interpretation) were also captured. These workflows were later translated into the ‘generalized function” layer of the AH. Using our boundary definition, previous work regarding medical records [34], and discussions with SMEs, 5 goals were developed to describe the purpose of treatment. These formed the “functional purpose’ layer of the AH. Whereas a purely biomedical treatment goal would be to “improve health,” concepts such as patient education and public safety were included in the scope of patient treatment.

After describing goals and processes, a list of concepts that linked these layers was developed with help from SMEs. This included articulating abstract concepts such as values and balances, and showing how system goals were mediated to perform functions. These concepts were translated into the “abstract function” layer of the AH.

The physical functions layer of the AH represented concepts, objects, and actors that were needed to perform the processes. The physical form represented details and attributes of the objects and actors that were relevant to the system processes. For example, the social status and severity of symptoms were relevant attributes of the patient.

## Results

**Figure 3:**
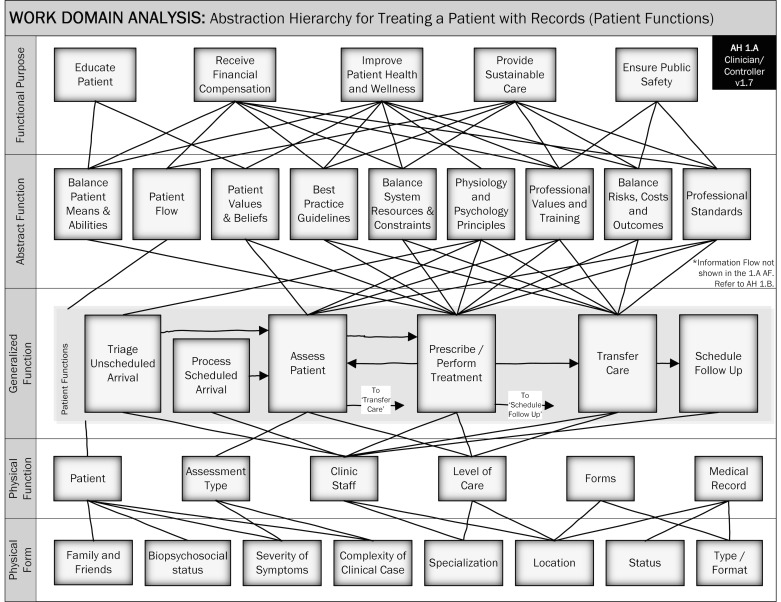
Abstraction Hierarchy 1.A, describing patient functions.

**Figure 4:**
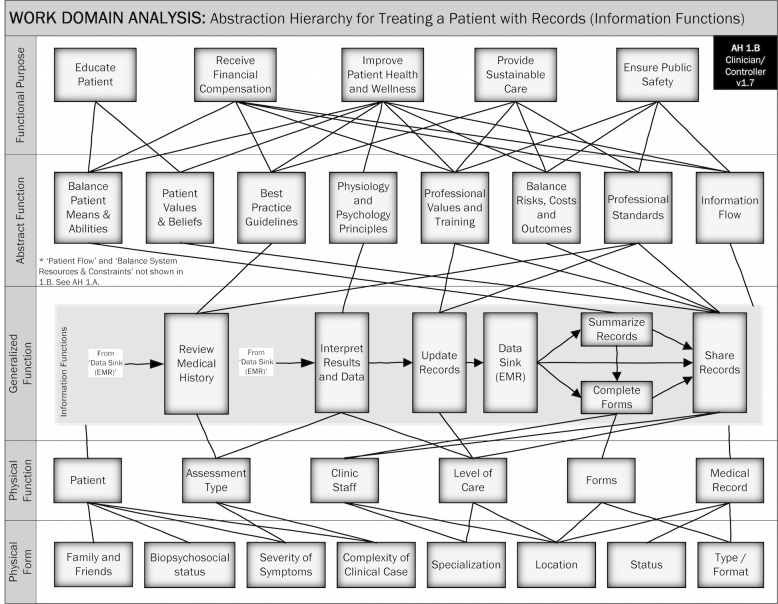
Abstraction Hierarchy 1.B, describing information functions.

The patient flows and information flows each were placed into separate views of the same AH (see Figures 3 and 4). Showing two views increased the readability of the hierarchy and allowed each type of process to be displayed separately. Other than the generalized functions and specifically noted omissions, all elements of the model are shown in each view.

### Functional Purpose and Treatment Goals

Five functional purposes were identified in the AH. The treatment purposes (eg, goals) included concepts of patient education, financial compensation, health improvement, sustainable care, and public safety. These goals are linked to abstract functions which represent constraints to be respected in achieving each goal. In some situations, each goal is met during treatment. In some situations, one goal may take priority over the other. For example, a patient with a communicable disease may need to be quarantined to ensure public safety at the expense of their individual wellness and freedom. However, the goal of the modeled system generally is to achieve all goals simultaneously outside of fringe cases. The details underlying these concepts were developed in consultation with SMEs.

#### Patient Education

As part of treating patients, clinicians aim to educate patients. This includes providing information about health conditions, treatments, and lifestyle. Educating patients is an important goal in their treatment since poor education or incorrect information can interfere with treatment and must be considered as a goal. For example, SMEs mentioned that some patients may not wish to be vaccinated based on individual patient beliefs about vaccines. In this context, the overall goal of treating a patient is a combination of education, improving their health, and ensuring public safety from communicable diseases.

As shown in the AH, during treatment, patient education is mediated by patient means and abilities (eg, patients who cannot afford physiotherapy might be educated about exercises instead of receiving a referral) and patient values (eg, not being willing to accept a certain treatment).

#### Financial Compensation

In Ontario, fee-for-service payments are provided by the Government, a third party insurance provider, or the patients themselves. In other situations, such as clinicians who are part of a FHT or physicians who work at a community health center, clinicians are salaried and employed by the Government to provide health care services and treat patients. Sometimes physicians are compensated through a combination of patient-capitation (eg, payment per patient per year), by the services provided and according to special bonuses for achieving specific care practices [23].

While treatment could be modeled altruistically, payment to clinicians impacts the treatment approach. As mentioned by SMEs, some doctors in the fee-for-service model adopt a “one visit, one problem” approach to maximize potential remuneration. Since this decision is influenced by financial remuneration and not driven by best practice or health outcomes, this concept is important to capture in the AH and show as a treatment goal that impacts clinical processes through abstract functions.

As shown in the AH, compensation is mediated by a patient’s resources (eg, ability to pay uncovered costs and fees), best practice guidelines (eg, government bonuses for specific additional interventions, which are based on best practice guidelines), patient flow (eg, volume and theoretical maximum billable time), system resources (eg, the government budget), professional values and training (eg, what services can be performed and opting to select strategies favoring maximum remuneration), balancing risks versus benefits (eg, determining whether receiving compensation for the treatment is worth any potential risks or benefits to the patient), and professional standards (eg, what actions are permitted, ethical, and appropriate).

#### Patient Health and Wellness

An obvious goal of patient treatment is to improve patient health and wellness. Patients who are not looking to improve their health or wellbeing (directly or indirectly) will not seek treatment. SMEs mentioned that sometimes patients seek assistance for social reasons and not for strictly medical reasons; the biopsychosocial nature of care accords services to patients who are isolated socially or are experiencing significant life challenges such as job loss or homelessness.

Improving patient health is mediated by patient resources (eg, financial ability to pay clinicians when required, afford drugs, or have social supports to support care), the ability to actually see the patient (eg, patient flow), patient values and beliefs (eg, willingness to accept recommendations), best practice guidelines, health system constraints (eg, scheduling constraints for referrals), physiology and psychology principles, professional values and training (eg, what treatment can be performed), and balancing the costs and benefits of a treatment plan.

#### Sustainable Care

In Ontario, clinicians need to select appropriate tests and treatments that support a sustainable health care system. Clinicians also need to avoid unnecessary procedures that are of limited clinical value. For example, SMEs described patients who request “fad” bloodwork, such as a vitamin test, without a clinical reason. Unlike the fully privatized health care systems, clinicians need to make treatment choices that respect the public purse and support a sustainable health care system by ensuring diagnostics are medically necessary. This type of conflict resolution is challenging [3] and is important to include as a constraint in treatment. Patients are not always able to receive the tests and treatments that they want because of limited health resources.

Sustainable care is moderated by best practice guidelines, patient flow (eg, volume and capacity), system constraints (eg, budgetary limits), professional values (eg, caring about the public purse), and professional standards of care (eg, guidelines).

#### Public Safety

Clinicians must place individual patient treatment into the context of public safety. Patients who are a danger to others, have communicable diseases, or could endanger their community in other ways (eg, poor eyesight in a senior citizen who drives) require interventions that are not necessarily in the patient’s best interest. For example, SMEs discussed that taking a senior citizen’s driver’s license may protect public safety, but may also result in social isolation and poor medical outcomes for the individual patient. Public safety is an important element to model in health care. Ensuring public safety is moderated by professional values and training, the balancing of risks versus outcomes, and professional standards. This also has an impact on many information flows, such as mandatory reporting requirements [25].

### Abstract Functions and Treatment Constraints

Abstract functions represent constraints that need to be respected during clinical processes (eg, generalized functions) to achieve the system’s treatment goals. These concepts were created in consultation with SMEs.

#### Patient Means and Abilities

Patient means (eg, financial and social) and abilities (eg, mental competency and self-care) need to be balanced and considered in their treatment. For example, SMEs mentioned that a physician will need to take a patient’s ability to pay for drugs into consideration when issuing a prescription or recommending physiotherapy. Likewise, an elderly patient’s access to peer groups and family would impact their ability to live at home or require homecare. This constraint influences treatment functions and plays a role in how medical records are processed (eg, looking up patient details and social circumstances) and summarized for sharing (eg, summarizing data for a referral).

#### Patient Values and Beliefs

Patients have varying worldviews and values that need to be understood and balanced during treatment. For example, SMEs mentioned that some religions would object to blood transfusions, some cultures will not tolerate birth control, some peer groups adhere to false information about vaccines, and some female patients may be uncomfortable with a male doctor performing certain medical procedures. This abstract concept plays a role in patient assessment and treatment procedures. Patient beliefs also may play a role in how information is shared with other providers based on patient perspective about privacy rules and regulations [35].

#### Best Practice Guidelines

Best Practice Guidelines suggest health screenings, preventative tests, and appropriate actions for patients with specific characteristics (eg, age and diagnosis). SMEs referred to guidelines that recommend specific treatment functions (eg, recommending a test), or specify that a patient be transferred to another level of care (eg, sending a patient to a stroke unit from the emergency room). Best practice guidelines have a significant impact on the review of medical history. The constraints on treatment that are associated with best practice guidelines are represented through this abstract function, but guideline documents were not included in the Physical Function of the AH in order to manage project scope.

#### Patient Flows

Patient flow is a representation of patients entering, moving through, and exiting the treatment process. Patient flow represents limits related to patient volume and throughput. Patient volume is an important constraint on the system, as the flow of the patient through the clinic and the health care system must be taken into consideration and is important for all generalized functions. Without capacity, treatment is not possible.

#### System Resources and Constraints

As a single-payer, publicly-funded health care system, health care dollars and resources in Ontario must be taken into consideration during treatment. Not all drugs or treatments are available, and some procedures have significant waiting lists due to insufficient system resources (eg, number of beds and number of surgeons). This abstract function describes a constraint in selecting treatment options for patients while achieving treatment goals.

#### Physiology and Psychology Principles

Human anatomy, physiology, and pathophysiology principles are important constraints to be considered during treatment. When patients are suffering from situations that are not strictly biomedical in nature (eg, social distress, isolation, and stress), psychological principles need to be taken into account. This abstract function helps describe constraints during triage, patient assessment, treatment, and transfer of care. From an information perspective, these principles are important when clinicians interpret results and data and update the medical record.

#### Professional Values and Training

Clinicians are not uniform in their decisions. As with patients, clinicians have worldviews, professional values, and priorities. For example, physicians may choose to see more patients in a day (eg, volume) and provide care to a large number of patients, or may choose to see fewer patients for full assessments to provide higher-quality care. Worldviews also may impact ethical decisions, such as valuing the public purse. A professional’s scope of practice, practice style, and set of priorities is based on training and personality characteristics. This abstract value system plays a role in assessing patients, performing treatments, and deciding when it is appropriate to transfer care. It also plays a role in a clinician’s interest in creating high-quality documentation that is above minimum standards. Patient and documentation processes are constrained by professional values and training.

#### Risks, Costs, and Outcomes

Whenever treatment is provided to a patient, there are risks, possible outcomes, and costs. If a clinician determines that the risk is high and the probability of a positive outcome is low, another treatment option may be selected. Similarly, a clinician may balance the health care costs of surgery for an arthritic patient versus a prescription, and make a treatment determination that is based on total costs, recovery periods, and quality of life. Risk balancing takes place in consultation with patients who describe their preferences and capabilities. In situations where patients pose a risk to public safety, a clinician must make an appropriate determination between risks and potential negative outcomes to the patient and public.

This abstract concept plays a role in assessments and treatments. Risks also are evaluated when choosing to transfer care. Information functions assist in determining risk.

#### Professional Standards

All clinicians are governed by professional associations and colleges. For example, physicians in Ontario are governed by the College of Physicians and Surgeons of Ontario (CPSO). The CPSO establishes specific conditions and training requirements for all physicians in Ontario. They have policies on medical records [24] and provide guidelines regarding reporting information to third parties [25]. The concept of professional standards constrains patient assessment; prescription and treatment; transfer of care; and maintaining, reviewing, updating, and sharing medical records. To manage the scope of the domain analysis, the standard documents were not included in the scope of the model and are not included in the physical function of the AH as well.

#### Information Flow

Information flow is a representation of information that enters the system and is used and stored in an EMR. Information flow is important in managing care and impacts decision making and timing. If information is not available when needed, it will affect many aspects of treatment. As an abstract concept, information flow is important through all information functions in the generalized function layer of the model. Information flow impacts financial compensation (eg, ability to bill and document encounters), patient health (eg, improved care quality through information), and public safety (eg, reporting mandatory information to appropriate authorities).

### Generalized Treatment Processes

The generalized functions represent the general processes in health care, as described in Figures 1 and 2. Each generalized function was linked to abstract function constraints that had to be respected to achieve the system goals and to the appropriate physical components of the processes.

### Physical Treatment Elements and Attributes

The physical functions layer of the AH represents concepts, objects, and actors that were needed to perform the processes modeled in the generalized functions. The physical form represents details and attributes of the objects and actors that are relevant to the system processes. Keeping in mind that the clinician is the system controller (and is not represented in the physical form), the relevant actors and objects in the AH include the patient, type of assessment, clinic staff, level of care, forms, and medical records.

#### Patient

The patient is obviously an important actor associated with all generalized functions. The patient’s attributes that are relevant in treatment include patient’s family and friends (eg, presence of social supports to facilitate treatment), the patient’s biopsychosocial status (eg, social circumstances such as employment and stressors), the severity of the patient’s symptoms or problems, and the complexity of the clinical case. The patient and their most important attributes are included in the model as they affect the entire treatment ecosystem.

#### Assessment Type

Different types of assessments are used. A physical exam would be detailed, whereas a 10-minute assessment would be problem-oriented. Other assessments may play the role of triage and refer a patient directly to the hospital (from primary care) or admit a patient (from the emergency room). Severity and complexity play roles in the type of assessment that will be used with the patient.

#### Clinic Staff

The clinical staff supports many processes. Depending on the specializations and location of the care delivery, resources may be greater or fewer. Larger clinics with multiple clinicians will have a larger support staff with specific roles and responsibilities. Smaller clinics with an individual doctor may only have a single support resource who plays a generalist role. The type of staff and their abilities varies according to location of the practice.

#### Forms

Many forms are employed to support the information processes during treatment. The location of the forms and their type (paper or electronic) are relevant attributes to the information flows and processes described in Figure 4.

#### Medical Record

Medical records support all the information flow processes. The location of the status (eg, availability), record type (eg, paper or electronic), and location of the system are relevant attributes.

## Discussion

### Comparisons With Other Models

Our WDA and AH is interesting because it describes patient treatment in the context of a complex biopsychosocial ecosystem (Figure 3) and patient treatment in an electronic health record context (Figure 4). Each view shows how the rich ecosystem system influences patient treatment and records management.

The view showing patient treatment flows is different compared with existing models in the literature; as an AH, the model can articulate complex ideas within the treatment ecology and is a formative reference model. No existing AH describes how treatment takes place with clinician-controllers and modern, Internet-enabled patients. As a macro-level view of patient treatment, our model is similar to a model of medication administration in home care, which facilitated an in-depth understanding of medication safety problems and analyzed medication errors [14].

The view showing records management flow is also unique in the literature. To our knowledge, there are no WDA in the literature that describe records management with a complex sociotechnical perspective. The results of this view could be very interesting to health information management professionals who are concerned with data quality, and to EMR developers trying to understand the work context of their users.

### Design Implications

WDAs and AHs are consumed during design by using the ecological interface design (EID) approach [11,36]. The AH can support system designers by properly articulating the ecosystem and clinician decision making in context. The model supports system thinking and can help articulate how changes may impact the ecosystem through linear and ripple effects [37]. Based on our work domain analysis, the decision support requirements for health care are becoming increasingly complex. The challenge for system engineers will be to determine how electronic systems could support, and not hinder, the treatment process. In addition, the analysis is a reminder that technology-centric solutions and implementations that do not take the larger health care ecology into consideration during the entire treatment process will likely fail to thrive. Creating a product that is compatible with the nuances that are described in the AH would be a competitive advantage.

### Limitations

Our AH is intended to be helpful, but it is not perfect. The model is limited to a clinician’s perspective and aims to provide a high-level overview of treatment. Obvious opportunities are present for a deeper analysis of the work domain in special areas. For example, complex nuances to medication, prescription, and administration have been simplified and abstracted in our model as “Prescribe and Perform Treatment.” It would be possible to do a more detailed WDA on this specific issue. For example, Lim et al developed a detailed analysis of medication administration in home care [14], and this could be performed in primary care. In this sense, our work is incomplete. In this same sense, the amount of modeling to be performed is infinite, and our hierarchy is a contextual overview that could serve as a blueprint for additional work.

One potential limitation of our work was the availability of SMEs and volunteers to validate the model in clinical practice. Though we interviewed several SMEs with different backgrounds and roles in the health care system, we did not adopt a formal grounded theory approach to our information gathering. Though the use of techniques such as grounded theory may have improved and formalized our qualitative data collection, formalized approaches are not standard practice for conducting CWAs and building AHs. In this sense, this limitation is not uncommon in the literature. Based on the concept of our WDA being a helpful, but not perfect, model, this is not a significant or unusual limitation.

### Future Work

The current AH describes patient treatment and takes a biopsychosocial perspective over a biomedical one. Taking a patient-centered perspective further, the AH could more formally incorporate aspects of SDM thinking. This would be compatible with the current work, as general qualities of treatment with SDM include deliberation with patients, an individualized approach, information exchange, involvement of multiple parties, finding middle ground, espousing mutual respect, developing patient education, encouraging patient participation, and following a process with stages [38]. Adopting SDM is a desirable approach to care; improved patient involvement in decision making can result in improved health outcomes, provide a better ethical framework for clinicians to deliver appropriate care and can improve the health system’s efficiency [39]. However, it is important to note that SDM is not always easy for clinicians to implement, and barriers exist to its use in patient care: in addition to requiring new time management strategies, it also might not apply to the patient’s characteristics or their clinical situation [40]. Thus, a goal would be to capture SDM and non SDM procedures, values, and concepts.

It would be interesting to compare SDM and non SDM perspectives with patient care. Inviting SMEs to comment and develop a similar AH could lead to an interesting comparison of work, as the current work does include an SDM expert in its development. Such a comparison could help to describe the perceptions and realities of what shared decision making is and how it is (or is not) incorporated in routine clinical care. The idea of drawing comparisons has previously been discussed [41].

Another interesting perspective about SDM is that it is a shared process between at least two actors; colloquially, SDM has been described as a dance between providers and patients [42]. Thus, developing a full perspective of SDM will require at least one other AH describing patients as a controller. Work by Rezai and Burns [13] could provide a good starting point for developing an AH from a patient perspective. Team perspectives to patient care modeled with SOCA [12] also could be helpful for understanding SDM in care teams comprised of family physicians, nurse practitioners, pharmacists, medical specialists, caregivers, and patients [40,43]. Generally, further work on this AH and line of inquiry could lead to interesting contributions to SDM research.

### Conclusions

Our AH links treatment goals, decision-making constraints, and task workflows. The model articulates the immense task complexity and nuanced user needs in today’s patient treatment by describing the system’s goal, abstract functions, workflows, and physical characteristics. The model can be used by system developers to improve systems by better supporting complex decision making in context. The model could support the development of EMRs that incorporate the cognitive processes associated with patient treatment by transferring the knowledge from our WDA into design concepts through EID. Currently, the hierarchy is a contextual overview of the treatment domain from a clinician’s perspective and additional models could further articulate depth and details in subdomains of the system.

## Abbreviations

CWA: cognitive work analysis
ConTA: control task analysis
CPSO: College of Physicians and Surgeons of Ontario
EID: ecological interface design EMR: electronic medical record
SME: subject matter expert
WDA: work domain analysis
SDM: shared decision making
